# The Protective Effects of EMF-LTE against DNA Double-Strand Break Damage In Vitro and In Vivo

**DOI:** 10.3390/ijms22105134

**Published:** 2021-05-12

**Authors:** Hee Jin, Kyuri Kim, Ga-Young Park, Minjeong Kim, Hae-June Lee, Sangbong Jeon, Ju Hwan Kim, Hak Rim Kim, Kyung-Min Lim, Yun-Sil Lee

**Affiliations:** 1 College of Pharmacy, Ewha Womans University, Seoul 03760, Korea; jinhee2848@naver.com (H.J.); kyuri@ewhain.net (K.K.); mwz11036@naver.com (G.-Y.P.); nabe37@naver.com (M.K.); 2 Division of Basic Radiation Bioscience, Korea Institute of Radiological & Medical Sciences, Seoul 03760, Korea; hjlee@kirams.re.kr; 3 Radio & Satellite Research Division, Electronics and Telecommunications Research Institute, Daejeon 34129, Korea; sbjeon@etri.re.kr; 4 Department of Pharmacology, College of Medicine, Dankook University, Chungnam 31116, Korea; cruse731@hanmail.net (J.H.K.); hrkim@dankook.ac.kr (H.R.K.)

**Keywords:** DNA damage, repair gene expression, electromagnetic waves, LTE, B16, HaCaT

## Abstract

With the rapid growth of the wireless communication industry, humans are extensively exposed to electromagnetic fields (EMF) comprised of radiofrequency (RF). The skin is considered the primary target of EMFs given its outermost location. Recent evidence suggests that extremely low frequency (ELF)-EMF can improve the efficacy of DNA repair in human cell-lines. However, the effects of EMF-RF on DNA damage remain unknown. Here, we investigated the impact of EMF-long term evolution (LTE, 1.762 GHz, 8 W/kg) irradiation on DNA double-strand break (DSB) using the murine melanoma cell line B16 and the human keratinocyte cell line HaCaT. EMF-LTE exposure alone did not affect cell viability or induce apoptosis or necrosis. In addition, DNA DSB damage, as determined by the neutral comet assay, was not induced by EMF-LTE irradiation. Of note, EMF-LTE exposure can attenuate the DNA DSB damage induced by physical and chemical DNA damaging agents (such as ionizing radiation (IR, 10 Gy) in HaCaT and B16 cells and bleomycin (BLM, 3 μM) in HaCaT cells and a human melanoma cell line MNT-1), suggesting that EMF-LTE promotes the repair of DNA DSB damage. The protective effect of EMF-LTE against DNA damage was further confirmed by attenuation of the DNA damage marker γ-H2AX after exposure to EMF-LTE in HaCaT and B16 cells. Most importantly, irradiation of EMF-LTE (1.76 GHz, 6 W/kg, 8 h/day) on mice in vivo for 4 weeks reduced the γ-H2AX level in the skin tissue, further supporting the protective effects of EMF-LTE against DNA DSB damage. Furthermore, p53, the master tumor-suppressor gene, was commonly upregulated by EMF-LTE irradiation in B16 and HaCaT cells. This finding suggests that p53 plays a role in the protective effect of EMF-LTE against DNA DSBs. Collectively, these results demonstrated that EMF-LTE might have a protective effect against DNA DSB damage in the skin, although further studies are necessary to understand its impact on human health.

## 1. Introduction

With the rapid growth of the electronic device industry, humans are extensively exposed to radiofrequency electromagnetic fields (RF-EMF) through numerous electronic devices and wireless communications [1,2,3]. Simultaneously, public concern over the adverse health effects of RF-EMF is escalating. Many prior studies have evaluated the harmful effects of EMFs on human health, including functional cell damage [4], genetic damage, neurological diseases, reproductive disorders, immune disorders, kidney damage, electromagnetic hypersensitivity, and leukemia [5,6,7,8,9,10]. However, these studies have been criticized for methodological limitations, unrealistic exposure levels of RF-EMF, or lack of in vivo evidence to substantiate the in vitro findings related to the hazards of RF-EMF [11]. In addition, RFs are widely used in medical devices for skin tightening and improvement of skin laxity and cellulite without significant adverse effects [11,12]. These discordant findings indicate the need to clarify the hazards of RF exposure to human health.

One of the most controversial impacts of RF exposure on human health is its effect on DNA damage. Many studies have found that RF radiation can cause DNA damage in human cells, such as the epithelial cells in the lens and human hair follicles [13,14]. In contrast, other studies have found no significant impact of RF exposure on DNA damage [15,16,17]. It seems that the impact of RF exposure on DNA damage is dependent on flux densities, frequencies, and exposure patterns of EMF [18]. Further complicating this issue, several studies have revealed contradicting effects of EMF on DNA repair genes involved in base excision repair (BER), nucleotide excision repair (NER), and non-homologous end-joining (NHEJ) pathways depending on cell type. Following co-exposure of 15 min field-on/15 min field-off of 0.50 mT 50 Hz ELF with morphine, the mRNA levels of Gadd45A (BER pathway), XRCC1, XRCC4, Ku80, Ku70, and LIG4 (NHEJ pathway) were down-regulated in SH-SY5Y [19]. Moreover, triple treatment with cisplatin, morphine, and 50 Hz ELF potentiated the down-regulation of genes involved in the NHEJ pathway, resulting in sensitization of cancer cells to DNA double-strand breaks (DSBs) induced by bleomycin. Interestingly, these effects were not observed in MCF-7 cells, a human breast cancer cell line. In contrast, it has been reported that ELF (50 Hz) can improve the efficiency of DNA repair in human cancer cell lines, protecting cells against heat-induced apoptosis [20]. However, it remains unclear whether RF-EMF exposure affects DNA repair or induces DNA damage.

The skin is the outermost layer of the body and is exposed directly to ionizing and non-ionizing radiation, such as gamma rays, ultraviolet rays, electromagnetic waves, and heat [21,22]. The impact of these radiations on the skin considerably varies from burn to photoaging depending on wavelength and energy [23]. Interestingly, radiation with long wavelengths (such as near-infrared and red light) can have beneficial effects such as accelerating wound healing [24] and DNA excision repair [25]. However, it remains unclear whether EMF-RF has protective effects against DNA damage in the skin.

In this study, we investigated the effects of EMF-LTE (1.762 GHz, 8 W/kg) on DNA DSBs in skin cells, a B16 murine melanoma cell line and a human keratinocyte cell line (HaCaT), using an in vitro LTE exposure system [26]. To confirm these effects in vivo, C57/BL6 mice were irradiated with EMF-LTE (1.76 GHz, 6 W/kg) for four weeks, and the skin tissues were examined for the DNA DSB marker γ-H2AX in an effort to elucidate the effects of EMF-LTE on DNA damage in the skin.

## 2. Results

### 2.1. Effects of Electromagnetic Field-Long Term Evolution (EMF-LTE) on Cell Viability, Proliferation, Apoptosis, and Necrosis of Human Keratinocyte Cell Line (HaCaT) and Murine Melanoma Cell Line (B16) Cells

**Figure 1:**
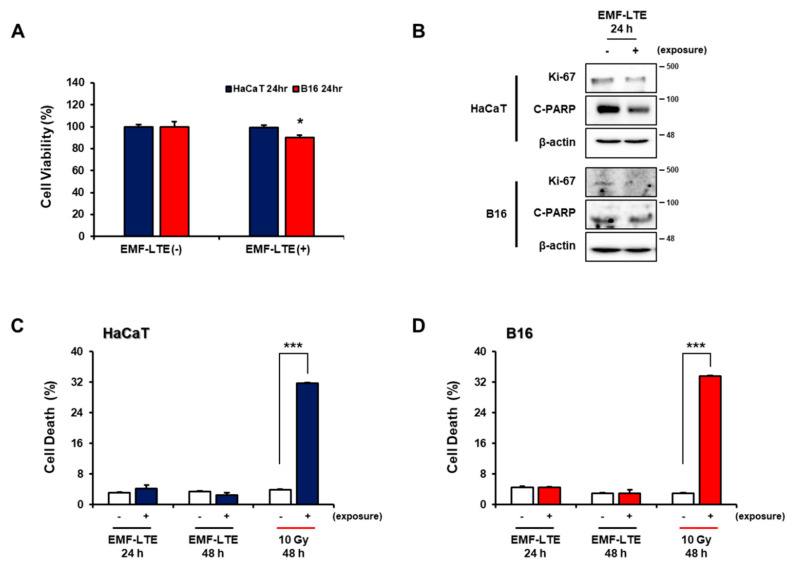
Effects of electromagnetic field-long term evolution (EMF-LTE) on cell viability, proliferation, and cell death of human keratinocyte cell line (HaCaT) and murine melanoma cell line (B16) cells. (**A**) MTT (3-(4,5-dimethylthiazol-2-yl)-2,5-diphenyltetrazolium bromide) assay in HaCaT cell and B16 cells was performed at 24 h after exposure to EMF-LTE. Each assay was performed in triplicate and in more than three independent experiments. * *p* < 0.05 vs. unexposed control (*t*-test). (**B**) Cell proliferation (Ki67) and apoptosis (C-PARP; Cleaved Poly ADP-ribose Polymerase) markers were examined with Western blot analysis. Representative photos are presented. (**C**) HaCaT and (**D**) B16 cells were treated with the indicated dose of EMF-LTE. After the indicated time, the cells were harvested and analyzed with flow cytometry after PI (propidium iodide) staining. Each assay was performed in triplicate and in more than three independent experiments. *** p < 0.001 vs. unexposed control (*t*-test).

First, to evaluate whether EMF-LTE exposure affects viability of the skin cell lines, EMF-LTE (1.762 GHz) was irradiated onto HaCaT and B16 at an intensity of 8 W/kg for 24 h, and cell viability was determined with a formazan-based viability assessment, the MTT (3-(4,5-dimethylthiazol-2-yl)-2,5-diphenyltetrazolium bromide) assay. Compared to the unexposed control, the cell viability in B16 was slightly reduced by EMF-LTE irradiation, while the HaCaT cells were unaffected (Figure 1A). Western blot analysis was conducted for markers of cell proliferation (Ki67) and apoptosis (C-PARP), along with FACS (Fluorescence-Activated Cell Sorting) analysis of cell necrosis with propidium iodide (PI) staining (Figure 1B–D). In both cell lines, EMF-LTE exposure slightly reduced the cell proliferation but did not cause apoptosis or cell necrosis, while the positive control, ionizing radiation (IR) exposure (10 Gy), significantly induced cell necrosis.

### 2.2. Effects of EMF-LTE on DNA Double-Strand Break Damage of HaCaT and B16 Cells

**Figure 2:**
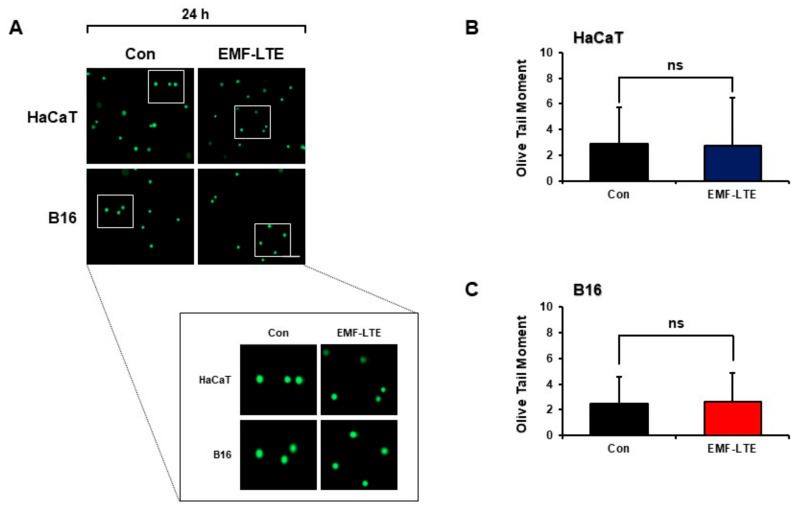
Effects of EMF-LTE on DNA DSB damage in HaCaT and B16 cells. (**A**) HaCaT and B16 cells were irradiated with EMF-LTE for 24 h, and the neutral comet assay was performed. Representative photos are presented. (**B**) (**C**) The olive tail moment was calculated using Comet 5.5 software. The scale bar indicates 50 μm (magnification: 5×). The data represent the mean ± SD. ns: not significant by paired sample *t*-test (*n* = 3).

To determine whether EMF-LTE can induce DNA double-strand break (DSB) damage, HaCaT and B16 cells irradiated with EMF-LTE for 24 h were subjected to the neutral comet assay. When the olive tail moment was calculated, there was no increase compared to that of the unexposed control cells (Figure 2A–C). The olive tail moment is a gel electrophoresis-based comet assay that measures DNA DSBs in individual cells. These results indicate that EMF-LTE alone did not induce DNA DSB damage in HaCaT or B16 cells.

### 2.3. Effect of EMF-LTE on Ionizing Radiation (IR)- or Bleomycin (BLM)-Induced DNA Double-Strand Break (DSB) Damage

**Figure 3:**
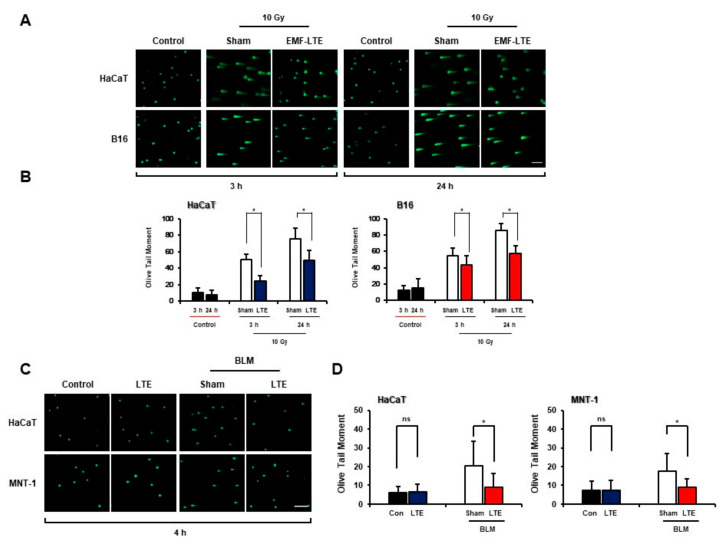
Effects of EMF-LTE on IR- or BLM-induced DNA DSB damage in HaCaT, B16, and human melanoma cells (MNT-1). (**A**) HaCaT and B16 cells were treated with EMF-LTE in combination with irradiation (10 Gy). The neutral comet assay was performed after the indicated time points. Representative photos are presented. (**B**) The olive tail moment was calculated using the Comet 5.5 software. (**C**) HaCaT cells and human melanoma MNT-1 cells were treated with EMF-LTE in combination with bleomycin (3 μM). The neutral comet assay was performed after 4 h. Representative photos are presented. (**D**) The olive tail moment was calculated using Comet 5.5 software. The scale bar indicates 50 μm (magnification: 5×). The sham control groups were only treated with radiation exposure or bleomycin. The data represent the mean ± SD. * *p* < 0.05, by *t*-test (*n* = 3). ns: not significant.

Previous studies have suggested that EMF-RF possesses tumor promotion effects [27] or potentiates the DNA damage induced by other stimuli such as reactive oxygen species and toxic drugs [19,28,29,30]. Therefore, to determine whether EMF-LTE affects the DNA DSB damage induced by DNA-damaging stimuli, skin cells were pre-treated with IR (10 Gy) and then exposed to EMF-LTE (8 W/kg) for 3 h or 24 h (Figure 3A). As a result, the smeared olive tail moments were significantly increased in IR-exposed cells. Remarkably, treatment with EMF-LTE after IR irradiation in HaCaT cells decreased the olive comet tail moments from 50.4 to 24.5 and from 75.5 to 49.7 at 3 and 24 h, respectively. In B16 cells, the comet tail moments were reduced from 54.2 to 43.7 and 85.4 to 57.6 at 3 and 24 h, respectively, after EMF-LTE treatment (Figure 3B). These results suggest that EMF-LTE promotes the repair of IR-induced DNA DSB damage. We also evaluated the effects of EMF-LTE exposure on DNA DSB damage induced by bleomycin (BLM), a genotoxic cytotoxic agent [31]. EMF-LTE was exposed to cells treated with 3 μM of BLM. Therefore, similar to IR, treatment with BLM alone dramatically increased the olive tail moments in HaCaT and MNT-1 cells, a human melanoma cells. However, when the cells were treated with both EMF-LTE and BLM, the comet tail moments were significantly reduced compared to those with BLM treatment alone (Figure 3C,D).

### 2.4. Effects of EMF-LTE on IR-Induced DNA DSB Damage Marker γ-H2AX in HaCaT and B16 Cells

**Figure 4:**
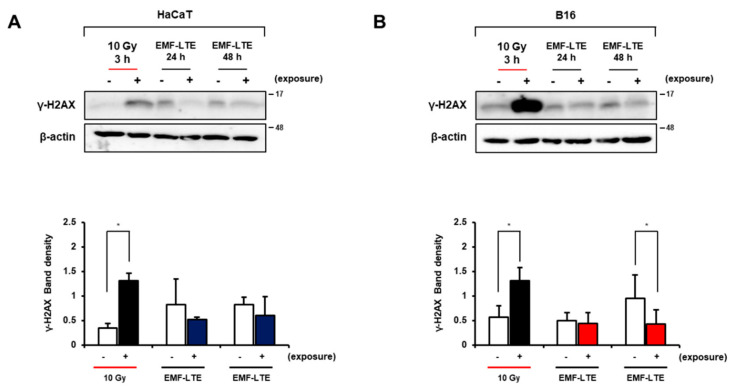
Effects of EMF-LTE on DNA DSB damage markers in HaCaT and B16 cells. The representative blotting images of γ-H2AX protein in (**A**) HaCaT and (**B**) B16 cells are presented (upper panel). Band density values were normalized to that of β-actin (lower panel). Graphs represent the mean ± SD of three independent experiments (* p < 0.05 vs. corresponding control, paired sample *t*-test).

**Figure 5:**
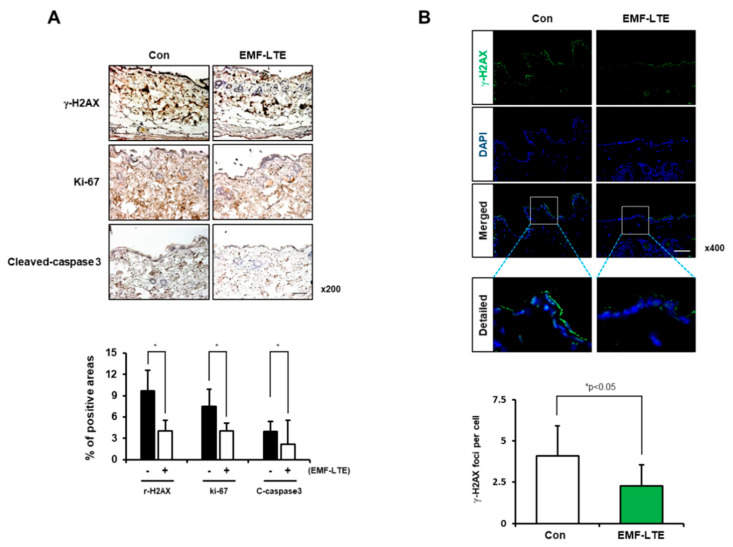
Effects of EMF-LTE on DNA DSB damage markers in mouse skin. (**A**) Immunohistochemistry of mouse skin tissue with antibodies for γ-H2AX, Ki67, and cleaved-caspase 3 was conducted. The percentage of positively stained cells per field is shown. Representative photos are presented. The scale bar indicates 50 μm (magnification: 200×). (**B**) Immunofluorescence staining of mouse skin tissue with anti-γ-H2AX antibody (green) after EMF-LTE. The nuclei were counterstained with DAPI (blue) (upper panel). Representative photos are presented. The scale bar indicates 50 μm (magnification: 400×). The number of γ-H2AX foci for cells in 30 fields was counted, as was the average number of foci per cell. Quantification was performed using Image J software. The data represent the mean ± SD. * p < 0.05 compared with the unexposed control group (*n* = 3).

To further confirm the effects of EMF-LTE exposure on DNA damage, the level of γ-H2AX, a DNA DSB damage marker, was determined in the skin cells. Western blot analysis revealed that the γ-H2AX level was significantly increased in HaCaT and B16 cells by 10 Gy IR irradiation. In contrast, EMF-LTE exposure did not increase the γ-H2AX level in either HaCaT or B16 cells (Figure 4A,B). Interestingly, compared to the untreated control, the γ-H2AX level was significantly attenuated by 48 h of EMF-LTE exposure in B16 cells, a trend similarly observed in HaCaT cells, although statistical significance was not achieved. Of note, the reduced level of γ-H2AX was confirmed in the skin tissue of EMF-LTE-exposed male C57/BL6 mice (1.76 GHz 6 W/kg, 8 h per day for 4 weeks) using immunohistochemistry and immunofluorescence staining (Figure 5A,B). These results suggest that DNA DSB damage in the skin tissue can be attenuated by EMF-LTE. Interestingly, reduced cell proliferation and apoptosis were observed in immunohistochemistry of Ki67 and cleaved caspase-3, in line with the findings in vitro.

### 2.5. Effects of EMF-LTE on the Expression Levels of DNA Repair Genes

**Figure 6:**
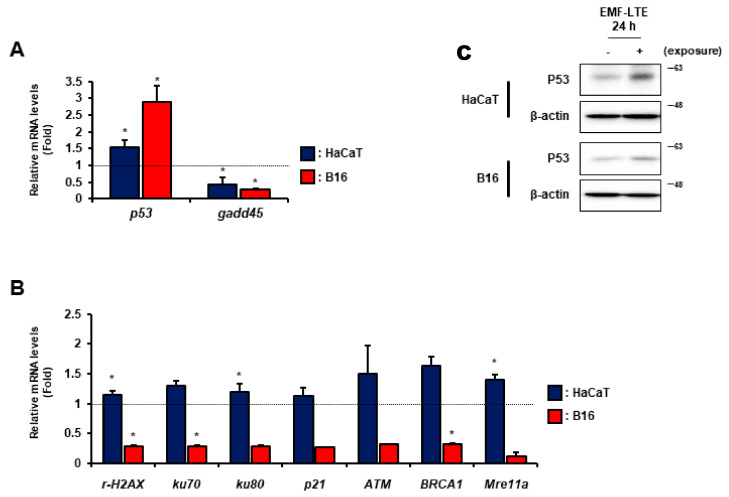
Effects of EMF-LTE on DNA repair genes in HaCaT and B16 cells. Real-time polymerase chain reaction (PCR) analysis to evaluate the relative mRNA levels of known DNA repair genes was performed in HaCaT (**A**) and B16 cells (**B**). Each mRNA expression was normalized to that of GAPDH (glyceraldehyde 3-phosphate dehydrogenase), which is an internal control gene. The data reflect the mean ± SD. Statistical significance was measured using the *t*-test. Fold change values were generated after normalization with average values of the unexposed control. (**C**) Western blot analysis for P53 was performed with β-actin as a loading control. Representative photos are presented. * *p* < 0.05.

To investigate the expression of DNA repair genes after EMF-LTE exposure, quantitative real-time polymerase chain reaction (qRT-PCR) analysis using both HaCaT and B16 cells was performed on nine DNA repair genes. The mRNA levels of the DNA damage sensor genes (*p53*, *γ-H2AX*, *ATM*, and *p21*), NER (*gadd45*), NHEJ (*ku70* and *ku80*), and HR (*BRCA1*, and *Mre11a*) genes were evaluated after EMF-LTE exposure for 24 h. The *p53* and *gadd45* genes were identified as genes commonly responsive to EMF-LTE in both cell lines. The *p53* level was upregulated, but *gadd45* was downregulated (Figure 6A,B). In contrast, the expression patterns of some genes were different between HaCaT and B16. In HaCaT cells, the EMF-LTE exposure significantly increased the expression levels of several genes, including *γ-H2AX*, *ku80*, and *Mre11a*, while *ku70*, *p21*, *ATM*, and *BRCA1* showed increasing trends. However, in B16 cells, all the mRNA levels investigated (except for *p53*) were significantly decreased or showed decreasing trends (Figure 6B). Of note, the increased P53 level was confirmed in both cell lines with Western blot analysis (Figure 6C).

## 3. Discussion

As telecommunication technology advances rapidly, human exposure to radiofrequency electromagnetic fields (RF-EMF) has become unavoidable. In particular, radiofrequency (RF), which has an EMF with a bandwidth of 3 kHz to 300 GHz, is widely used in everyday life [3]. Therefore, there is increasing concern over the potential carcinogenic effects of RF exposure [32]. Previous studies purporting the carcinogenic effects of RF-EMF have suggested two hypotheses. RF-EMF can act as either a tumor-promoting agent or as a co-carcinogen to trigger indirect DNA damage [33,34,35]. The tumor-promoting effects of 1960 MHz EMF-RF in ethylnitrosourea-treated mice have been reported, indicating that the greater is the RF-EMF exposure, the greater are the size and incidence of tumors [27]. RF-EMF emitted by mobile phones was classified in 2010 as a “possible carcinogen” by the International Agency for Research on Cancer (IARC) [3]. However, there have been contradicting reports regarding the carcinogenic effects of RF-EMF. Studzinski et al. reported that RF-EMF (2450 MHz 5 and 15 mW/cm^2^ for 9 months) can increase benzopyrene induced-skin cancer in mice [36]. However, Chagnaud et al. did not observe such an effect in rats after irradiation with 900 MHz microwaves 55 or 200 μW/cm^2^ (75 and 270 mW/kg average whole-body SAR(Specific Absorption Rate), 2 h daily for 2 weeks) [37]. Moreover, an in vivo study confirmed that RF-EMF exposure to fetal mice did not increase ethylnitrosourea-induced DNA damages in the brain, liver, or lung [32]. These findings demonstrate that further studies are needed to elucidate the effects of RF-EMF on DNA damage.

To investigate how EMF-LTE (1.762 GHz, 8 W/kg) affects skin cells, we compared the effects of EMF-LTE and unexposed controls on cell viability, proliferation, and death. We showed that exposure to EMF-LTE for 24 h at 8 W/kg slightly decreased cell viability in B16 cells, while it had no effect on HaCaT cells. We also showed that EMF-LTE reduced cell proliferation but did not cause apoptosis or cell necrosis. To confirm the DNA damage seen with EMF-LTE (1.762 GHz, 8 W/kg), a comet assay was performed at neutral pH. The comet assay did not demonstrate an increase in olive tail moments compared to that of the unexposed control cells. Choi et al. reported that exposure of various cell lines to 1.7 GHz LTE RF-EMF at 1, 2 W/kg for 72 h decreased cellular viability and cell proliferation [38]. These results are consistent with those of our study, which showed anti-proliferation effects of EMF-LTE. However, in contrast to our study, Choi et al. found that 1.7 GHz LTE RF-EMF (1, 2 W/kg, 72 h) exposure did not induce DNA DSB or apoptotic cell death in various cell lines [38]. Unlike these results, we showed that EMF-LTE (1.762 GHz, 8 W/kg) decreased DNA DSB protein r-H2AX and apoptotic protein (C-PARP, cleaved caspase 3) both in vitro and in vivo. This might stem from the difference in cell lines and exposure conditions (exposure time or intensity) across studies.

We demonstrated that EMF-LTE exposure can attenuate spontaneous and genotoxic agent-induced DNA DSB damage, as shown by a decreased γ-H2AX protein level and attenuation of IR- or BLM-induced olive comet tail moments. Promotion of DNA repair responses by EMF is not a new concept. Nikolova et al. demonstrated that ELF and RF-EMF exposure increased cell cycle regulatory “growth arrest DNA damage-inducible” GADD45 genes along with pro-apoptotic genes in embryonic stem cell-derived neural progenitor cells [39]. Interestingly, Kim et al. showed that visible red light has protective effects against ultraviolet B (UVB)-induced DNA damage in human dermal fibroblasts. This phenomenon is presumably mediated by enhancement of GADD45A-mediated DNA repair activity [25], which suggests an important role of GADD45 in the effects of EMF on DNA damage.

We examined the expression of DNA repair genes including *p53*, *gadd45*, *γ-H2AX*, *ku80*, *Mre11a*, *p21*, *ATM*, and *BRCA1*. In both HaCaT and B16 cell lines, the *p53* gene level was upregulated, while the *gadd45* gene level was downregulated. Upregulation of the p53 protein in cells occurs under various environmental stresses, and EMF was reported to induce p53 expression [40]. Gadd45 is a gene regulated by p53 that plays an essential role in the NER pathway [41]. In the same study, although the p53 level was increased by EMF-LTE, the gadd45 level decreased. This finding differed from recent reports that showed that exposure to RF-EMF (1.71 GHz) or ELF-EMF (50 Hz) increased *gadd45* mRNA level in mouse embryonic neuronal stem cells [39]. This discrepancy can be explained by differences in cell lines and exposure conditions (exposure time or intensity), which must be further evaluated.

The gene expression of *γ-H2AX*, *ku80*, *Mre11a*, *p21*, *ATM*, and *BRCA1* were different between B16 and HaCaT cells. These findings suggest that EMF-LTE exposure has different effects on various repair genes. However, the origin of these differences is not known. One possibility is that species differences accounted for the divergent results, since B16 is a murine melanoma cell line and HaCaT is a human keratinocyte cell line. Another possibility is the status of transformation as B16 cells are tumor cells, while HaCaT cells are non-transformed. Therefore, the difference in DNA repair systems might have have been activated in response to EMF-LTE irradiation. Ultimately, this requires further investigation.

In summary, although more detailed mechanistic studies are needed, this study demonstrated that DNA DSB damage can be attenuated by EMF-LTE irradiation in the skin both in vitro and in vivo. These findings provide an important line of evidence illuminating the potentially beneficial effects of EMF-LTE.

## 4. Materials and Methods

### 4.1. In Vitro Exposure to EMF-LTE, IR, and Bleomycin

Cells in 60 mm cell culture dishes were placed in an exposure chamber and exposed to 1.762 MHz EMF-LTE (SAR; 8 W/kg) that was developed and reported previously [26,38] for the indicated time. The exposure level and schedule were controlled by a control unit. The maximum input power was 60 W, and the input signal was fed through a conical antenna with broadband characteristics. The external dimensions of the exposure system were 843 mm × 825 mm × 315 mm. The exposure system was specifically designed to control the environmental conditions, including ventilation, humidity, and temperature. To maintain CO_2_ density and humidity inside the chamber, gas from an incubator was circulated throughout. A pump circulating water was used to protect against increase in culture medium temperature during RF exposure by circulating the water from the cooling system. The SAR measurement was preliminarily performed using a Luxtron 812 fiber optic thermometer from Luxtron Corporation with a thermal resolution of 0.1 °C according to the Equation (1) below:

(1) $$\mathrm{SAR}=\mathrm{Cp}\;\frac{\Delta T}{\Delta t}\approx \mathrm{Cp}\frac{dT}{dt}$$

The measurement probes were located at nine points inside a petri dish. The exposure system was warmed for at least 30 min for equilibration prior to EMF-LTE exposure.

For the positive controls, the cells were cultured in 96-well plates or dishes and exposed to gamma radiation doses (10 Gy as a single dose) generated by a ^137^Cs gamma-ray source (MDS Nordion, Ottawa, ON, Canada) at a dose rate of 5 Gy/min. For bleomycin (Santa Cruz Biotechnology, Dallas, TX, USA) exposure, the cells in 60 mm cell culture dishes were treated with bleomycin (3 μM) for 4 h. The combination experiments comprised three conditions: cells were exposed to an EMF-LTE (8 W/kg) for an indicated time immediately after irradiation with gamma rays, cells were exposed to EMF-LTE for 4 h in the presence of bleomycin, and sham exposure was performed in the cell culture incubator.

### 4.2. Cell Culture

The human keratinocyte cell line HaCaT (German Cancer Research Center, Heidelberg, Germany), Mus musculus skin melanoma cell line B16 (Korea cell Line Bank, Seoul, Korea), and human skin melanoma cell line MNT-1 (ATCC, Manassas, VA, USA) were cultured in Dulbecco’s modified Eagle’s medium (DMEM) supplemented with 10% fetal bovine serum and 1% penicillin-streptomycin at 37 °C in a humidified 5% CO_2_ incubator.

### 4.3. MTT (3-(4,5-Dimethylthiazol-2-yl)-2,5-diphenyltetrazolium bromide) Assay

Cell viability was evaluated by MTT assay in 96-well plates. Cells were seeded in a 96-well plate at a concentration of 1 × 10^4^ cells/well. After exposure, the cells were incubated with 100 μL 0.5 mg/mL 3-(4,5-dimethylthiazol-2-yl)-2,5-diphenyltetrazolium bromide (Sigma, St. Louis, MO, USA) for 2 h. The absorbance was determined colorimetrically at 540 nm using an ELISA microplate reader (TECAN, Mannedorf, Switzerland).

### 4.4. Flow Cytometry Analysis

Analysis of cell death was performed with propidium iodide (Sigma, St. Louis, MO, USA) staining and flow cytometry. A density of 2.5 × 10^5^ cells was seeded in 60 mm cell culture dishes and incubated. Total cells were collected by trypsinization and centrifugation at 1300 rpm for 3 min. The cells were washed with phosphate-buffered saline (PBS) and stained with propidium iodide (PI) 0.5 µg/mL. The samples were analyzed using a FACS Calibur (BD Biosciences, San Jose, CA, USA), and the cell data were analyzed by BD Cell Quest Pro Software (version 6.0, BD Biosciences, San Jose, CA, USA).

### 4.5. Neutral Comet Assay

Neutral Comet assays were performed under neutral conditions as previously described [42], with minor modifications. Briefly, HaCaT, B16, and MNT-1 cell suspensions were mixed with low-melting agarose (LMagarose) at 1 × 10^4^ cells/mL. The cells were then pipetted onto microscope slides and maintained at 4 °C for 10 min to solidify. The slides were immersed in chilled lysis solution (Trevigen, Gaithersburg, MD, USA) at 4 °C for 2 h in the dark and then in TBE buffer (45 mM Tris-borate, 1 mM EDTA) for 10 min. Electrophoresis was performed at 25 V for 20 min. The slides were washed with distilled water, fixed at 70% EtOH for 5 min, and stained with 20 μL SYBR (Trevigen, Gaithersburg, MD, USA). Images of the comets were captured under a Zeiss fluorescent microscope (Cal Zeiss, Oberkohen, Germany) at 5× magnification. For each sample, a minimum of 50 comets was obtained, and the olive tail moment (tail DNA (%) × (tail mean—head mean)) was analyzed using the Comet Assay Software (Komet 5.5, Andor Technologies, Abingdon, UK). Each assay was performed in triplicate and in at least three independent experiments.

### 4.6. Immunoblotting

After EMF-LTE exposure, equal amounts of protein were dissolved in lysis buffer. Protein concentration was determined by the Bradford method (Bio-Rad, Hercules, CA, USA). The samples were boiled for 5 min, and the proteins were separated by 15% sodium dodecyl sulfate polyacrylamide gel electrophoresis (SDS-PAGE). The separated proteins were transferred to nitrocellulose membranes. After blocking with 5% skim milk in phosphate-buffered saline with Tween-20 (PBS-T), the membranes were incubated with antibodies for KI-67 (DAKO, CA, USA), C-PARP (Cell Signaling Technology, Danvers, MA, USA), P53 (Santa Cruz Biotechnology, Dallas, TX, USA), γ-H2AX antibody (EMD Millipore, Billerica, MA, USA), and β-Actin (Santa Cruz Biotechnology) overnight at 4 °C. The cells were then washed with 1X PBS-T and incubated with horseradish peroxidase (HRP)-conjugated anti-mouse secondary antibody (Santa Cruz Biotechnology). The HRP activity was measured using enhanced chemiluminescence (EzWestLumi, Taito-ku, Tokyo, Japan). Protein band intensity was visualized on ChemiDoc (Bio-Rad) and quantified using Image J software 1.45 (National Institutes of Health, Bethesda, MD, USA).

### 4.7. Animals

All procedures were approved by the Animal Care and Use Committees of Korea Institute of Radiological and Medical Sciences (KIRAMS) Institutional Animal Care and Use Committee (IACUC permit number: KIRAMS2019-0023) and were performed in accordance with the relevant guidelines. We used methods as described previously [43,44] with minor modifications. Briefly, animal exposures were performed in a reverberation chamber with dimensions of 2295 × 2293 × 1470 mm^3^ and a one-mode stirrer. An RF LTE signal source with a center frequency of 1.76 GHz, bandwidth of 20 MHz, and QPSK modulation was employed for experiments. This input signal was amplified by a high-power amplifier (PCS60WHPA_CW; Kortcom) and injected into an antenna inside the reverberation chamber (the maximum input power allowed was 60 W). Real-time monitoring of this input power (i.e., the output of the high-power amplifier) was performed with a power meter (Keysight, N1912A) through a 20 dB directional coupler (Keysight, 778D). The field uniformity of the reverberation chamber was measured at 24 points inside the working volume. An isotropic field probe (HI-6005; ETS-Lindgren) was employed for electric field measurements. The uniformity averaged over 1 min was evaluated according to a previous report [45]. The field distribution was well within ± 2 dB inside the working volume. The detailed description of the chamber evaluation was as reported previously [46]. The SAR simulations were based on the mouse anatomical model (IT’IS Foundations, Male-OF1-Mouse model). The reverberation chamber was placed in the animal facility, and the ventilation, temperature, and humidity were controlled. Male C57/BL6 mice were obtained from Orient Biotech Co. Ltd. and were randomly assigned to two groups (6 animals per group). The animals were exposed to the 1.76 GHz RF-EMF according to the following schedule: Whole-body average SAR 6 W/kg, 8 h/day, 5 days/week, for 4 weeks. The mice of the sham-exposure group were placed inside the chambers without RF-EMF signals during the same period. During RF exposure, the air temperature inside the test area was maintained at 20 ± 3 °C, and the mice were able to move freely. Rectal body temperature was measured before and immediately after RF-EMF exposure and never increased by more than 0.5 °C.

### 4.8. Immunohistochemistry (IHC) and Immunofluorescence Staining (IF)

For immunohistochemistry, skin samples were cut into 5 μm sections and were sequentially rehydrated with a descending graded series of ethanol. For antigen retrieval, the slides were placed in citric acid buffer (Abcam, Cambridge, UK) and heated at 100 °C for 10 min. Immunohistochemical staining was performed using anti-γ-H2AX (1:100 dilution; EMD Millipore), Ki-67 (1:200, DAKO), and cleaved-caspase 3 (1:200, Cell Signaling Technology) at 4 °C overnight. The slides were incubated with an avidin-biotin peroxidase complex (ABC kit, Vector Laboratories, CA, USA) and developed using 3,3′-diaminobenzidine tetrachloride (DAB; Zymed Laboratories, CA, USA). For immunofluorescence staining, skin samples were processed using the same procedure as for immunohistochemistry until primary antibody incubation. After incubation with primary antibodies, the samples were immediately washed in PBS two times and incubated with Alexa-488-conjugated anti-mouse (Invitrogen, Carlsbad, CA, USA) for 1 h at room temperature. The nucleus was counterstained with 4’, 6-diamidino-2-phenylindole (DAPI) (Sigma-Aldrich). All immunofluorescence images were captured by a Zeiss Apotome (Cal Zeiss).

### 4.9. RNA Isolation and Quantitative Real-Time Polymerase Chain Reaction (qRT-PCR)

Total RNA was isolated from the sample using TRIzol^®^ reagent (Qiazen, Valencia, CA, USA). RNA purity and concentration were measured with a Nanodrop. The RNA was reverse transcribed using a ReverTra Ace^®^ qRT-PCR Kit (TOYOBO, Kita-ku, Osaka, Japan) following the manufacturer’s protocol. PCR was performed to assess expression of the candidate genes using primers designed for mRNA sequences. In addition, mRNA expression was assessed using real-time PCR with a SYBR Green PCR Master Mix kit (Bioline USA Inc., Taunton, MA, USA) and CFX96 Touch™ Real-Time PCR Detection System (Biorad, USA). The 2^−ΔΔ*C*t^ method was used to analyze the relative changes in gene expression from real-time quantitative PCR experiments. GAPDH was used as an internal control gene. The primer sequences for qRT-PCR are listed in Table 1.

### 4.10. Statistical Analysis

Data were analyzed using GraphPad Prism 5.0 (GraphPad Software Inc., San Diego, CA, USA). Statistical differences compared with the control group were determined by Student’s *t*-test; *p* values < 0.05 were considered significant.

## Figures and Tables

**Table 1:** Gene sequences used in quantitative real-time polymerase chain reaction (qRT-PCR) analysis.

	Gene Name	Sequence (Human)	Sequence (Mouse)
1	*p53*	Forward: AGGCCTTGGAACTCAAGGAT	Forward: CACGTACTCTCCTCCCCTCAAT
		Reverse: TGAGTCAGGCCCTTCTGTCT	Reverse: AACTGCACAGGGCACGTCTT
2	*r-H2AX*	Forward: TGGAAAGGGTCAGGGAACG	Forward: AACGACGAGGAGCTCAACAAGC
		Reverse: GACTTGTGCTGGTATCTGGGTG	Reverse: TGGCGCTGCTCTTCTTGGGCA
3	*ku80*	Forward: GTTCTAAAGGTCTTTGCAGCAAGA	Forward: AACATGGTCGCCATCGTCCGAT
		Reverse: AAAAGCCACGCCGACTTGAGGA	Reverse: CCGCAAGTCTTCCATGAAAGGC
5	*ku70*	Forward: GGTTTCAAGCCGTTGGTACTGC	Forward: GCAGTCTACTCCTGCCTAGTGA
		Reverse: CTCCAGACACTTGATGAGCAGAG	Reverse: ACCTGGCTCATCAAACCGCTTC
7	*gadd45*	Forward: CGTTTTGCTGCGAGAACGAC	Forward: CCTGGAGGAAGTGCTCAGCAAG
		Reverse: GAACCCATTGATCCATGTAG	Reverse: GTCGTCTTCGTCAGCAGCCAG
8	*p21*	Forward: GCTTCATGCCAGCTACTTCC	Forward: TCGCTGTCTTGCACTCTGGTGT
		Reverse: CCCTTCAAAGTGCCATCTGT	Reverse: CCAATCTGCGCTTGGAGTGATAG
9	*ATM*	Forward: TGTTCCAGGACACGAAGGGAGA	Forward: CCAAGATGGCAGTGAACCAGAC
		Reverse: CAGGGTTCTCAGCACTATGGGA	Reverse: ATGCTGGACAGCTATGGTGGAG
10	*BRCA1*	Forward: CTGAAGACTGCTCAGGGCTATC	Forward: CGAGGAAATGGCAACTTGCCTAG
		Reverse: AGGGTAGCTGTTAGAAGGCTGG	Reverse: TCACTCTGCGAGCAGTCTTCAG
13	*Mre11a*	Forward: CAGCAACCAACAAAGGAAGAGGC	Forward: CCCTGACAATCCTAAGGTGACC
		Reverse: GAGTTCCTGCTACGGGTAGAAG	Reverse: CGTAGTCGGATAAGAGGCTTCC
14	*gapdh*	Forward: TTCGACAGTCAGCCGCATCTTCTT	Forward: ACTGTGGTCATGAGCCCTTC
		Reverse: GCCCAATACGACCAAATCCGTTGA	Reverse: GGGTGTGAACCACGAGAAAT

## Data Availability

The data presented in this study are available on the request to the corresponding author.

## Abbreviations

EMFs	Electromagnetic fields
LTE	Long term evolution
EMF-LTE	EMF-long term evolution
EMR	Electromagnetic radiation
IR	Ionizing radiation
NIR	Non-Ionizing radiation
ELF	Extremely low frequency
RF	Radio frequency
BER	Base excision repair
NER	Nucleotide excision repair
NHEJ	Non-homologous end-joining
MTT	[3-(4,5-Dimethylthiazol-2-yl)-2,5-diphenyltetrazolium bromide]
